# Consenting for intraoperative use of animal- and human-derived products: a case of the blind leading the blind?

**DOI:** 10.1308/rcsann.2025.0063

**Published:** 2025-10-01

**Authors:** MA Jama, MF Bath, JM Wohlgemut, K Bateman, MEJ Wise, NA Yassin

**Affiliations:** ^1^ Sheffield Teaching Hospitals NHS Foundation Trust, UK; ^2^ University of Birmingham, UK; ^3^ University of Cambridge, UK; ^4^ NHS Greater Glasgow and Clyde, UK; ^5^ Cardiff and Vale University Health Board, UK; ^6^ Central and North West London NHS Foundation Trust, UK; ^7^ University Hospitals Birmingham NHS Foundation Trust, UK

## Introduction

Growing cultural diversity and an increasing societal awareness of environmental changes have led to recent shifts in the lifestyle and dietary habits of the global population. Indeed, in the United Kingdom (UK), eating meat is no longer considered the norm and we have seen an increase of both veganism and vegetarianism, with around three million people in the UK now adhering to such lifestyles.^1^ Migration patterns have also led to increased religious and cultural diversity among the population that we, as surgeons, look after, and these groups may hold varied spiritual and moral reservations against the usage of (certain) animal products. Indeed, it is now estimated that up to 40% of the UK population has some form of limitation on their dietary intake or in the general use of animal products.^2^

With such changing acceptance towards the usage of animal- or human-derived products (AHDP), their inadvertent use in healthcare could lead to the potential for emotional or psychological harm for patients, or even the refusal of medical treatment.^3,4^ The usage of these AHDPs is very common in medicine, with at least 36 of the 100 most prescribed drugs in the UK deemed unsuitable for vegetarians.^5^ However, the literature reports poor labelling of these products, which leads to limited knowledge among healthcare professionals and risks poor counselling and consent practices.^2,5,6^ Surgical products are no exception to this.

Common medications provided to surgical patients, such as parenteral nutrition or enteral feeds, can often contain AHDP.^7–9^ In addition, AHDP form a constituent part of many surgical intraoperative products, such as mesh implants, suture materials or haemostatic agents.^8,10–12^ The intraoperative use of such materials poses the additional problem that their use is often irreversible; it can certainly be argued that specific consent for the use of such materials is essential, especially in a post-Montgomery era.^13^

## Challenges to consent

To better understand the current landscape for consent practices surrounding AHDP, we surveyed a wide range of surgeons from multiple general surgery subspecialties from across the UK. The results showed that, in those surveyed, there appears to be a lack of awareness of the products which do and do not contain AHDP, with just over 8% of respondents correctly identifying, for example, which haemostatic agents contained AHDP, and these percentages were even lower for meshes and suture materials. Perhaps more worrying, the majority of respondents (93%) stated that they rarely or never asked their patients about dietary lifestyles, or about religious or spiritual beliefs (85%). For many, this was a topic they had never considered or thought about, and they considered their own lack of knowledge as the greatest barrier to consent. These results demonstrate that the navigation of AHDP currently appears to be unfamiliar territory for many surgeons.

Although rulings by religious leaders on the use of AHDP are available for all major religious groups within the UK, it remains important to realise that individual beliefs might vary.^9,14^ Willingness to accept the use of AHDP might also depend on the availability of reasonable alternatives and the urgency of the surgical issue.^14,15^ Comparable variability in acceptance of AHDP can also be expected from vegan and vegetarian patients, with reported cases of vegan patients refusing to accept life-sustaining interventions that involve the use of AHDP.^16^ It becomes infeasible for generalisable recommendations to be created categorising every religious, spiritual, or moral choice.

In an already constrained healthcare system such as the National Health Service, ensuring an efficient preoperative period is essential.^17,18^ Although necessary, discussing the possibility for the unexpected use of haemostatic agents or non-biological alternatives, for example, will add significant time to the consent process, becoming an additional barrier for the majority of surgeons.^10^ Currently, UK regulations require pharmaceutical companies to include an ingredients list; however, it is not required to state whether products are suitable for vegetarians or label AHDP explicitly.^19^ This makes it difficult to ascertain whether AHDP are present - can surgeons be reasonably expected to investigate this for each product? Especially in time-sensitive emergencies, this could prove to be challenging. The potential refusal of AHDP also poses an additional ethical dilemma: how far would hospitals be expected to accommodate these wishes? It might not be financially feasible to purchase and stock the non-AHDP-containing alternatives.

**Figure 1:**
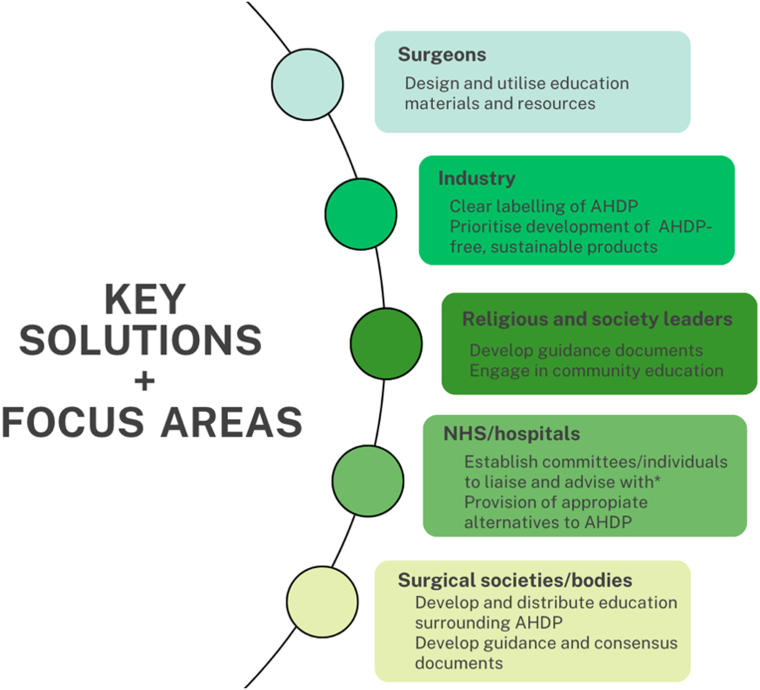
Suggested solutions and future areas of focus for the intraoperative use of animal- and human-derived products (AHDP)

Looking further afield, there are limited guidelines available globally from surgical societies worldwide on the use of intraoperative AHDP and how this should be incorporated into the consenting process. Although there are select countries with religious majorities that are affected by these dietary limitations in which clear labelling of these products has been established, it would appear that this is by no means the new status quo.^20^ For improved standards of care and consent, it is vital that new interventions and policies are devised by relevant healthcare, religious and lifestyle groups alike to provide clearer support and guidance on this important topic (Figure 1).

## Conclusions

The use of AHDP is an important area to consider in our surgical practice, reflecting the beliefs and wellbeing of the diverse populations we serve. Although there is no one-size-fits-all answer to consenting for AHDP, knowledge gaps are certainly present among surgeons and this forms a clear target for intervention. Accurate and transparent dialogue with patients remains a vital part of good surgical practice, even if ultimately the patient cannot be offered or guaranteed non-AHDP materials, and such discussions should be had early in the consent process.^21^ Informed decisions and consent must remain the main objective, as opposed to facilitating all dietary and religious needs; ensuring the utilisation of available information and resources, and engaging with patient groups in education will improve this, empowering patients in this often-challenging topic. The surgical community must take the lead in ensuring such changes are enacted to allow patients the opportunity to receive the best and most appropriate personalised care, which caters to both their medical needs and overall wellbeing.
